# Aberrant Cyclin D1 splicing in cancer: from molecular mechanism to therapeutic modulation

**DOI:** 10.1038/s41419-023-05763-7

**Published:** 2023-04-06

**Authors:** Jing Wang, Wei Su, Taotao Zhang, Shasha Zhang, Huiwen Lei, Fengdie Ma, Maoning Shi, Wenjing Shi, Xiaodong Xie, Cuixia Di

**Affiliations:** 1 School of Basic Medical Sciences, Lanzhou University, Lanzhou, 730000 China; 2 Bio-Medical Research Center, Institute of Modern Physics, Chinese Academy of Sciences, Lanzhou, 730000 China; 3 Key Laboratory of Heavy Ion Radiation Biology and Medicine of Chinese Academy of Sciences, Lanzhou, 730000 China; 4 College of Life Sciences, University of Chinese Academy of Sciences, Beijing, 101408 China

**Keywords:** Cancer, Molecular biology

## Abstract

Cyclin D1 (CCND1), a crucial mediator of cell cycle progression, possesses many mutation types with different mutation frequencies in human cancers. The G870A mutation is the most common mutation in *CCND1*, which produces two isoforms: full-length CCND1a and divergent C-terminal CCND1b. The dysregulation of the CCND1 isoforms is associated with multiple human cancers. Exploring the molecular mechanism of CCND1 isoforms has offer new insight for cancer treatment. On this basis, the alterations of *CCND1* gene are described, including amplification, overexpression, and mutation, especially the G870A mutation. Subsequently, we review the characteristics of CCND1 isoforms caused by G870A mutation. Additionally, we summarize cis-regulatory elements, trans-acting factors, and the splice mutation involved in splicing regulation of CCND1. Furthermore, we highlight the function of CCND1 isoforms in cell cycle, invasion, and metastasis in cancers. Importantly, the clinical role of CCND1 isoforms is also discussed, particularly concerning prognosis, chemotherapy, and radiotherapy. Last, emphasis is given to the corrective strategies that modulate the cancerous CCND1 isoforms. Thus, it is highlighting significance of aberrant isoforms of CCND1 as targets for cancer therapy.

## Facts

- The G870A mutation is the most common mutation in *CCND1*, which produces two isoforms: CCND1a and CCND1b.
- CCND1 isoforms are involved in tumor growth and progression by regulating cell cycle, invasion, and metastasis.
- CCND1 isoforms are associated with disease risk and clinical outcome in cancers, and can be used to predict cancer risk, clinical prognosis, or treatment response.
- Some antisense oligonucleotides or small interfering RNA can target tumor carrying CCND1b for treatment.

## Open questions

- CCND1 isoforms caused by G870A mutation that play a vital role in promoting the malignant phenotype of cancer, and what is the mechanism of its phenotype generation?
- Can CCND1b be exploited as a prognostic marker of cancers to make more accurate diagnosis, and supervise the response to treatment in patients with cancer?
- Various therapeutic strategies are available for targeting CCND1b, but what is the most effective therapy for cancer?

## Introduction

Cyclin D1 (CCND1), a crucial mediator of cell cycle progression, is the major cyclin involved in transition of cells from the G1 to S phase and plays a vital role in the pathogenesis of cancer [1, 2]. The amplification and/or overexpression of *CCND1* have frequently been found in a variety of cancers [3–14]. There are a large number of mutations in *CCND1*, which are closely related to the occurrence, development, prognosis, and treatment of cancers [15–21]. There are four major mutation types of *CCND1*, including missense mutation, truncating mutation, inframe mutation, and splice mutation [15]. The G870A mutation is the most common splice mutation in *CCND1*, and is associated with the risk, prognosis, and treatment of multiple cancers [20, 22, 23]. The conventional CCND1 consists of five distinct exons and has been studied extensively [24]. The G870A mutation produces two CCND1 isoforms by alternative splicing: full-length CCND1a and divergent C-terminal CCND1b [24, 25]. Recent studies have reported that CCND1a and CCND1b had equivalent function in regulating the cell cycle [26–29]. Zeng et al. found that both CCND1a and CCND1b can promote cell cycle progression and lead to cell proliferation, which may contribute to the potential oncogenic role of CCND1 isoforms in liver cancer Huh-7 and LO2 cell lines in vitro [26]. Interestingly, several studies have shown that CCND1b has different functions from full-length CCND1a [30, 31]. CCND1a can accelerate cell proliferation by promoting cell cycle progression, while CCND1b may inhibit cell cycle progression to prevent cell proliferation [31–35]. This unique activity of CCND1b is particularly significant in cancer treatment [36]. A great deal of study has indicated that dysregulated expression of CCND1 isoforms affect the multiple hallmarks of cancer [27, 37, 38]. For example, CCND1b could promote invasion and metastasis of breast cancer in a CCND1a-independent manner [38]. However, CCND1a conferred the resistance of cancer cells to DNA damage therapy by inducing DNA damage response (DDR) [39]. CCND1a and CCND1b play an essential role in modulating the switch between cell proliferation and death. Hence, this review summarizes the alterations of *CCND1* gene, including amplification, overexpression, and mutation, especially the G870A mutation. Subsequently, the characteristics of CCND1 isoforms caused by G870A mutation are described. Additionally, we summarize cis-regulatory elements, trans-acting factors, and the splice mutation involved in splicing regulation of CCND1. Furthermore, we highlight the function of CCND1 isoforms in cell cycle, invasion, and metastasis in cancers. Importantly, the clinical role of CCND1 isoforms is also discussed, particularly concerning prognosis, chemotherapy, and radiotherapy. Last, emphasis is given to the corrective strategies that modulate the cancerous CCND1 isoforms. Thus, our elucidation of CCND1 isoforms from different angles will contribute to better understanding the significance of CCND1 isoforms as a biomarker for cancer and targets for future therapeutic strategies.

## Alterations of *CCND1* gene

### Amplification and overexpression of *CCND1* gene

Amplification and overexpression are the most common mechanisms of alteration [40]. Gene amplification is related to the overexpression of oncogenes in cancers [41]. *CCND1* is located on chromosome 11q13, consists of 5 exons and 4 introns [42–46]. It was discovered in 1991 and is highly expressed in multiple cancers [43]. Its dysregulation may cause abnormal cell proliferation and contribute to the development of cancer [47, 48]. The amplification and/or overexpression of *CCND1* have frequently been found in a variety of cancers [3–14]. It was reported that approximately 15–20% of breast cancer contained the amplification of *CCND1* [49–51]. While the overexpression of CCND1 was observed in more than 50% of breast cancer [42, 52–55]. This indicates that although *CCND1* amplification is closely related to the overexpression of CCND1, the overexpression of CCND1 is not always secondary to gene amplification, and other mechanisms contribute to CCND1 overexpression. Amplification and overexpression of *CCND1* preferentially occurred in estrogen receptor (ER)-positive breast cancer [56–59]. Notably, the amplification of *CCND1* is related to the poor prognosis of ER-positive breast cancer and ER-positive tamoxifen-treated breast cancer [4, 60–62]. Nevertheless, the prognostic value of CCND1 overexpression is still controversial in breast cancer. Most studies showed that CCND1 overexpression could be considered as a marker of good prognosis in breast cancer [63–66]. However, some studies suggested that CCND1 overexpression was a poor prognostic marker in breast cancer [56, 67, 68]. In head and neck squamous cell carcinoma, the amplification and overexpression of *CCND1* were related to poor prognosis [69–72]. Interestingly, Kyomoto et al. found that *CCND1* amplification was a more effective prognostic marker than its overexpression in human head and neck squamous cell carcinoma [72]. Moreover, Miyamoto et al. demonstrated that *CCND1* amplification in oral cancer is a more reliable prognostic marker than CCND1 overexpression [73]. In addition, the amplification and overexpression of *CCND1* are considered to be related to poor prognosis in multiple cancers, including gastric cancer [5], esophageal cancer [7], colorectal cancer [10], thyroid papillary cancer [12], pancreatic cancer [13], cholangiocarcinoma [14], cervical cancer [74]. Currently, studies mainly focus on the relationship between the amplification and overexpression of *CCND1* and prognosis. In the future, we still need further investigation to fully clarify the role of the amplification and overexpression of *CCND1* in cancers, and to make use of its prognosis and predict the value of biomarkers.

### Mutation of *CCND1* gene

**Fig. 1:**
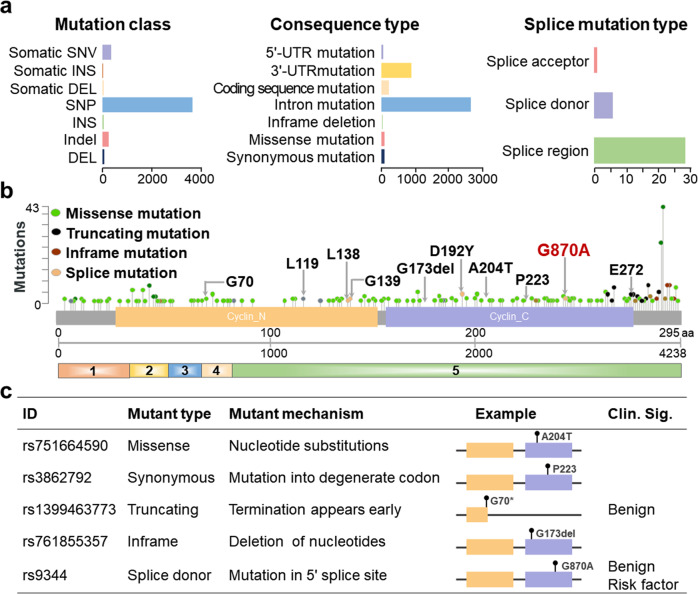
General characteristic of *CCND1* mutation. **a** The classification of *CCND1* mutations is based on different mutation types. **b** CCND1 is composed of two domains (N-terminal of cyclin_N and C-terminal of cyclin_C). The main mutation types of *CCND1* include missense mutations, truncating mutations, inframe mutations, and splice mutations. **c** Different mutation types are caused by different mechanisms and their clinical significance.

Currently, more than 4000 mutations are discovered in *CCND1* by the Catalogue of Somatic Mutations in Cancer and dbSNP databases [75, 76] (Fig. 1a). According to the mutation type of consequence, the major mutations in *CCND1* include synonymous mutation, missense mutation, intron mutation, coding sequence mutation, and 3′-UTR mutation. There are four major mutation types of *CCND1* based on the cBioPortal database, including missense mutation, truncating mutation, inframe mutation, and splice mutation [15]. The mutations are shown in Fig. 1b based on different mutation types. Missense mutation cause single amino acid substitutions [77]. Truncating mutation leads to protein truncation, which is closely related to many genetic diseases [78, 79]. Splice donor mutation are caused by mutations occurring at the 5′ splice site [24]. Moreover, synonymous mutation does not result in changes in amino acids due to the presence of degenerate codons [80]. Thus, different *CCND1* mutations are caused by diverse mechanisms and are related to the pathogenicity and risk of diseases (Fig. 1c).

According to The cBioPortal for Cancer Genomics database analysis of samples with mutation data in pan-cancer (TCGA PanCancer Atlas Studies), the mutation frequency of *CCND1* in patient is 6% [81, 82]. Plenty of studies have shown that the G870A mutation is the most common splice mutation in *CCND1*, and is associated with the risk, prognosis, and treatment of multiple cancers [20, 22, 23]. The G870A mutation has been determined to be related to the risk of a variety of cancers such as breast cancer [83], liver cancer [84], colorectal cancer [85], bladder cancer [86], endometrial cancer [87], esophageal and gastric cardiac cancer [88]. Table 1 summarizes the association between the G870A mutation of *CCND1* and 17 different cancers. Akhter et al. performed a meta-analysis of 18 published studies to clarify the connection between the G870A mutation and breast cancer risk by increasing statistical power and ultimately found that the AA genotype of G870A mutation was connected with an increased risk of breast cancer [83]. The association between the G870A mutation and cancer risk has been found in a greater part of cancers except for cervical cancer, esophageal squamous cell carcinoma, and prostate cancer [89–91]. Therefore, the G870A mutation of *CCND1* may be a key risk factor for cancers. In addition, other mutations of *CCND1* have been studied, such as rs614367, rs498136, and rs7177 [92, 93]. In the future, we need to further study the role of these mutations in cancers.

**Table 1:** The meta-analysis studies of *CCND1* G870A mutation in cancers.

Cancer	Case	Control	Number	Result	Ref
Breast cancer	12137	12309	18	Elevated risk for AA in Caucasian	[83]
Liver cancer	1018	1297	6	Association with liver cancer in Caucasians	[84]
Colorectal cancer	7276	9667	27	Elevated risk for A allele	[85]
Bladder cancer	3153	3670	9	Association with bladder cancer in Asian	[86]
Cervical cancer	1665	2511	5	No association with cervical cancer in Caucasian	[89]
Prostate cancer	3,820	3,825	5	No association with prostate cancer	[91]
Leukemia	227	218	3	Association with leukemia in allele, homozygote, recessive model	[205]
Brain cancer	2079	3690	9	Association with brain cancer in dominant, recessive, allele model	[206]
Nasopharyngeal cancer	844	1164	6	Association with nasopharyngeal carcinoma in Caucasians	[207]
	5338	6204	23	Elevated risk for A allele in Asian	[208]
Glioma	690	1014	4	Association with glioma in allele, dominant, codominant model	[209]
Esophageal cancer	2111	3232	11	Association with esophageal cancer in allele, codominant, recessive model	[210]
	2080	2833	7	No association with esophageal squamous cell carcinoma	[90]
Oral cancer	1377	1512	8	Association with oral cancer in Asian	[211]
Head and neck cancer	3761	3834	17	Association with head and neck cancer for smoking carriers of A allele and AA genotype	[212]
Non-hodgkin lymphoma	5357	4529	4	Association with non-Hodgkin lymphoma	[213]
Lung cancer	5162	5350	10	Elevated risk for A allele	[214]

## CCND1 isoforms caused by G870A mutation

**Fig. 2:**
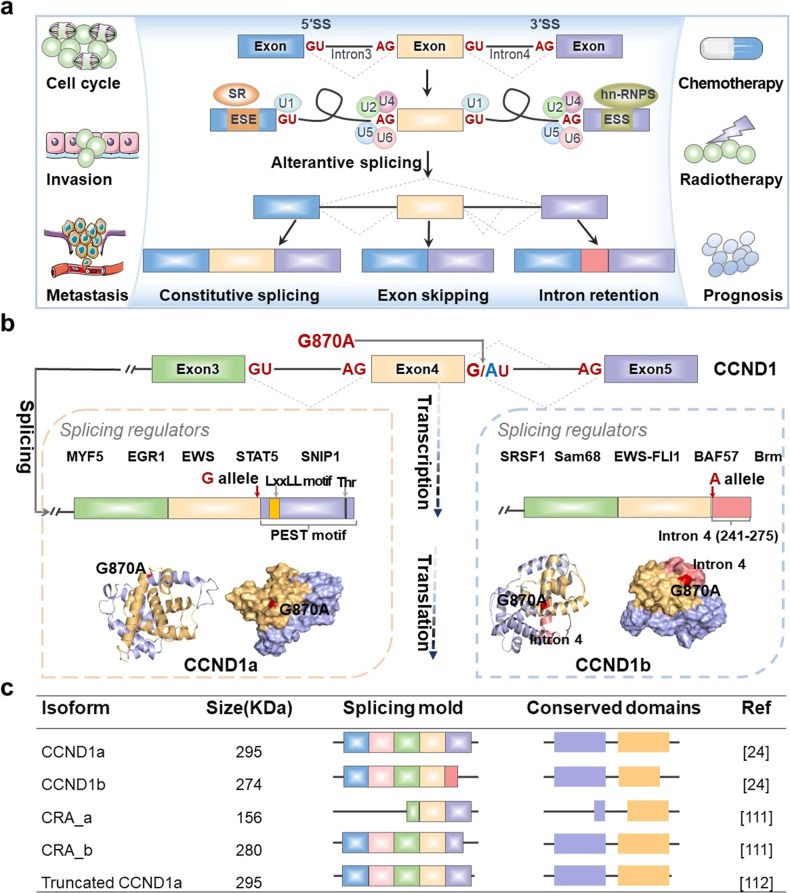
Regulation of G870A mutation on CCND1 pre-mRNA splicing and splicing isoforms of CCND1. **a** Alternative splicing and the effect of aberrant alternative splicing on cancer. The Figure shows some examples of cancer-specific alternative splicing events that contribute to the formation of distinct hallmarks of cancer. **b** The splice mutation of G870A occurs at the classical splice sites of exon 4 and intron 4. Carrying the G allele produces CCND1a. Carrying A allele produces CCND1b. In addition, the combination of cis-regulatory elements and trans-acting factors affects splice site selection. The protein structures of CCND1a and CCND1b are significantly different, and the mutation site is indicated by red. **c** General structure characteristics of CCND1 isoforms.

Alternative splicing produces multiple mRNA and protein isoforms by differential selection of splicing sites in precursor (pre)-mRNA (Fig. 2a) [94]. Splicing regulation plays a very important biological function, and aberrant splicing is one of the major causes of human cancer [95, 96]. A growing body of evidence has shown that aberrant splicing is widespread in cancers and plays a crucial role in cell cycle, invasion, metastasis, clinical prognosis, chemotherapy, and radiotherapy (Fig. 2a) [37, 48, 97–99]. The splice mutation is associated with multiple cancers and plays a crucial role in the regulation of pre-mRNA splicing [100–102]. The G870A mutation has been identified as a crucial splice mutation contributing to the production of CCND1a and CCND1b (Fig. 2b). Comstock et al. demonstrated that G870A mutation was associated with CCND1b by cloning the intron 4 sequences containing either the G or A allele [103]. It is generally recognized that carrying the G allele creates an optimal splicing donor site, resulting in a CCND1 transcript containing all exons (CCND1a). However, carrying the A allele may lead to abnormal splicing events, allowing partial intron 4 to remain and exon 5 to be removed, resulting in a CCND1b splice product. Several studies indicated that the A allele of G870A is preferentially related to the production of CCND1b [24, 25, 104]. CCND1a consists of five exons encoding a DNA sequence of 888 bp, which encodes a protein constituted of 295 amino acids. CCND1b is derived from a splice variant that fails to cleave at the intron 4 junction of exon 4 of CCND1 pre-mRNA to produce intron 4 retention. Since intron 4 contains a translation stop codon, CCND1b encodes a protein that produces 275 amino acids lacking exon 5 [48, 105]. There is a PEST sequence near the C-terminal of CCND1a, which is rich in Pro, Glu, Ser, and Thr residues. These residues play an important role in protein degradation [106]. In addition, the LxxLL motif is present on CCND1 and considered involving in the recruitment of SRC-1 [107]. The CCND1b protein has a completely divergent C-terminal domain, lacking the PEST motif and residues (Thr-286) that control nuclear export and protein stability [48, 108, 109]. Therefore, CCND1b was predicted to be a more stable constitutive nuclear protein with enhanced ability to modulate CDK activity and cell cycle progression. Loss of the LxxLL motif and changes in nuclear localization may alter the transcriptional activity of CCND1b [48]. We also simulate the protein structures of CCND1a and CCND1b using Swiss-Model software (Fig. 2b). It is obvious that the protein structure of CCND1b is different from CCND1a, which may lead to a special role for CCND1b in cancer. In addition, other isoforms encoded by CCND1 have been discovered in different cell types, such as CRA-a and CRA-b (Fig. 2c) [110]. Wiestner et al. have certified that CCND1 containing point mutations produced premature polyadenylation signals, leading to the production of CCND1a with truncated 3′-UTR that can significantly increase the carcinogenicity and worsen the clinical course in patients with mantle cell lymphoma [111].

## Regulation of CCND1 isoforms production

### Cis-regulatory elements

Cis-regulatory elements are short nucleotide motifs (∼5–15 base pairs) that bind to trans-acting factors and affect pre-mRNA splicing [112]. Cis-regulatory regions can present in exons or introns and act as an enhancer or silencer of splicing, specifically controlling alternative splicing by activating or inhibiting the use of adjacent splice sites [113]. The promoter region of *CCND1* has a variety of potential cis-regulatory elements that can bind to ATF/CREB, STAT5, STAT3, EGFR, EGR1, and AP1 which are important for pre-mRNA splicing and transcriptional activation of CCND1a in vitro [114–118]. Kang et al. found that a cis-regulatory element between −153 and −134 on *CCND1* promoter could bind to the EGR1 transcription factor to regulate CCND1a transcription in vitro and in vivo [117]. Moreover, a cis-regulatory element spanning −144 to −104 of *CCND1* promoter can enhance the transcription of CCND1a by binding to TGFα-induced EGR1 [119]. The cis-regulatory elements of *CCND1* promoters −58 [114], −954 [118], and −674 to −261 [115] region combined with trans-acting factors are also involved in the regulation of CCND1a transcription in vitro. Therefore, the above cis-regulatory elements can be combined with the corresponding trans-acting factors to regulate CCND1 pre-mRNA splicing.

### Trans-acting factors

Trans-acting factors are proteins that recognize or bind to *cis*-regulatory elements and participate in the formation of splicing regulatory networks, including serine/arginine-rich (SR) proteins, heterogeneous nuclear ribonucleoproteins, and some transcription factors [120]. Through the research on RNA binding protein Sam68, Paronetto et al. found that Sam68 was recruited to CCND1 and interacted with the proximal region of intron 4 to regulate its affinity for CCND1 intron 4, thereby stimulating the increase of CCND1b transcript in human prostate cancer PC3 cell line in vitro [121]. Moreover, the interaction of splice factor SRSF1 with BAF57/SMARCE1 mediated the mechanical stress-induced alternative splicing, producing CCND1b isoform in vitro [122]. Since alternative splicing is coupled with the transcription process, it is found that transcription factors also affected splice selection. Sanchez et al. demonstrated that EWS-FLI1 favored CCND1b expression by reducing the elongation rate, while EWS favored CCND1a expression in breast cancer MCF-7 and rhabdomyosarcoma A673 cell lines in vitro [28]. Additionally, BAF57 (SMARCE1) [122], MYF5 [123], SNIP1 [124], Brm (SMARCA2) [125], and other regulatory factors had also been reported to be involved in the splicing of CCND1 and summarized in Table 2.

**Table 2:** Trans-acting factors involved in CCND1 splicing regulation.

Regulation methods		Mechanism	CCND1a/b	Vitro/Vivo	Cell line	Ref
RNA binding proteins	Sam68	Regulate affinity for CCND1 intron 4	CCND1b	Vitro	PC3	[121]
	SRSF1 (ASF/SF2)	Affect splicing process by mechanical strains	CCND1b	Vitro	Mc3t3-E1/HaCaT	[122]
	MYF5	Mediate post-transcriptional mechanism	CCND1a	Vitro	C2C12	[123]
Transcription factors	ATF-2/CREB	Bind CCND1a -58 region	CCND1a	Vitro	MCF-7	[114]
	STAT5	Bind CCND1a -674 to -261 region	CCND1a	Vitro	NIH3T3/F-36P	[115]
	STAT3	Combine with CCND1 promoter region	CCND1a	Vitro	CNE1	[116]
	EGFR	Combine with CCND1 promoter region	CCND1a	Vitro	CNE1	[116]
	EGR1	Integrate promoter -153 to -134 region	CCND1a	Vitro/Vivo	INS-1	[117]
	AP-1	Combine with cis-regulatory elements (-954)	CCND1a	Vitro	Mv1.Lu/JEG-3	[118]
	EWS-FLI1	Influence transcription by elongation rate	CCND1b	Vitro	A673/MCF-7	[28]
	EWS	Alter transcription process by elongation rate	CCND1a	Vitro	A673/MCF-7	[28]
	SNIP1	Recruit RNA processing factor U2AF65	CCND1a	Vitro	U-2 OS/293T/HeLa	[124]
Others	BAF57 (SMARCE1)	Affect splicing process by mechanical strains	CCND1b	Vitro	Mc3t3-E1/HaCaT	[122]
	Brm (SMARCA2)	Affect splice sites and elongation rate	CCND1b	Vitro	C33A/MCF-7/Caco2/HeLa	[125]
	mTOR signal	Affect target of rapamycin (mTOR) activation	CCND1a	Vitro	LNCaP	[215]

### *CCND1* splice mutation

Mutation of the splice region is associated with multiple cancers and plays a crucial role in the regulation of pre-mRNA splicing [100, 101]. It is generally believed that splice mutation result in recognition of ectopic splice sites through pre-mRNA spliceosome, thereby changing splicing patterns, such as exon jumping and intron retention, and finally modulating the risks of cancer development and outcome [48, 126]. Table 3 summarizes some splice mutations of *CCND1* that may regulate pre-mRNA splicing of CCND1. A total of 36 mutations are found as splice mutations of *CCND1*, such as rs9344 (G870A), rs367683590, rs1268871232 (G139), rs1565224976, and rs201012923. In addition, the indel, somatic single nucleotide variant, somatic insertion, and somatic deletion are found to be related to splice mutation of *CCND1*. At present, it has been relatively clear that the G870A mutation regulates the splicing of CCND1 pre-mRNA [71, 72]. Figure 2b illustrates the process of G870A mutation regulating the splicing of CCND1 pre-mRNA. This mutation occurs at the boundary of intron 4/exon 5, which is located at the classical splicing donor site. It can regulate the production of CCND1a and CCND1b by interacting with cis-regulatory elements and trans-acting factors. Although cells carrying the A genotype tended to increase the production of transcript b, transcript a (CCND1a) can still be detected [127, 128]. This indicates that other factors may also affect the splicing of CCND1. However, the relationship between other factors and CCND1 splicing is still unclear and needs further investigation.

**Table 3:** Some splice mutations regulate splicing of CCND1.

Mutation ID	Class	Consequence type	Location	Splice mutation type
rs9344	SNP	Synonymous mutation	c.723G>A	Splice donor mutation
rs367683590	SNP	Synonymous mutation	c.412C>T	Splice region mutation
rs1268871232	SNP	Missense mutation	c.417A>C	Splice region mutation
rs1565224976	SNP	Missense mutation	c.199G>A	Splice region mutation
rs201012923	SNP	Missense mutation	c.577G>A	Splice region mutation
rs759345822	SNP	Missense mutation	c.197A>G/C	Splice region mutation
rs777225097	SNP	Missense mutation	c.722C>T	Splice region mutation
rs1424359226	SNP	Intron mutation	c.414+5C>G	Splice donor mutation
rs1339178943	SNP	Intron mutation	c.199-4G>A	Splice region mutation
rs1343339113	SNP	Intron mutation	c.198+8G>A	Splice region mutation
rs1398886316	SNP	Intron mutation	c.723+7G>A	Splice region mutation
rs377200375	SNP	Intron mutation	c.574+7C>T	Splice region mutation
rs571153521	SNP	Intron mutation	c.724-6T>C	Splice region mutation
rs758963834	SNP	Intron mutation	c.199-5C>A/T	Splice region mutation
rs776761881	SNP	Intron mutation	c.575-5C>G	Splice region mutation
rs1240440953	SNP	Intron mutation	c.415-5C>T	Splice region mutation
rs1420743674	SNP	Intron mutation	c.199-8C>T	Splice region mutation
rs1565072692	SNP	Intron mutation	c.575-6C>T	Splice region mutation
rs762325000	SNP	Intron mutation	c.415-8G>A	Splice region mutation
rs764630402	SNP	Intron mutation	c.724-4T>C/G	Splice region mutation
rs1456525574	SNP	Intron mutation	c.198+6G>A	splice donor mutation
rs374405138	SNP	Intron mutation	c.198+4C>T	Splice donor mutation
rs752676953	SNP	Intron mutation	c.198+5G>T	Splice donor mutation
rs1347517841	Indel	Inframe deletion	c.410TGCdel	Splice region mutation
rs1357027771	Indel	Intron mutation	c.575-19CTdel	Splice region mutation
rs751867946	Indel	Intron mutation	c.724-13CTdel/dup	Splice region mutation
rs763182769	Indel	Intron mutation	c.724-23_724-14dup	Splice region mutation
COSV99919240	Somatic SNV	Intron mutation	c.574G>T	Splice region mutation
COSV99919416	Somatic SNV	Intron mutation	c.723+1G>T	Splice donor mutation
COSV57120138	Somatic SNV	Intron mutation	c.415-1G>A	Splice acceptor mutation
COSV99919696	Somatic SNV	Intron mutation	c.575-3C>G	Splice region mutation
COSV57118864	Somatic SNV	Coding sequence mutation	c.723G>A	Splice region mutation
COSV57123050	Somatic SNV	Coding sequence mutation	c.199G>C	Splice region mutation
COSV99919217	Somatic SNV	Coding sequence mutation	c.726C>A	Splice region mutation
COSV99919589	Somatic insertion	Intron mutation	c.724-13_724-12insCT	Splice region mutation
COSV57123143	Somatic deletion	Intron mutation	c.575-14_575-13del	Splice region mutation

## The function of CCND1 isoforms in cancer

### Cell cycle

**Fig. 3:**
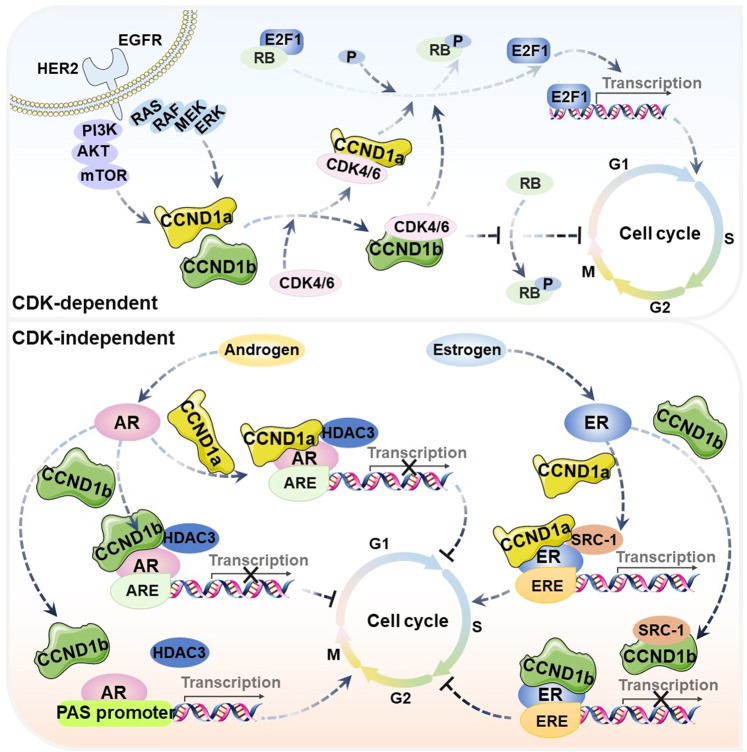
Cell cycle regulated by CCND1 isoforms. In the CDK-dependent mechanism, CCND1 isoforms combine with CDK4/6 to form the CCND1a/b-CDK4/6 complex. This complex is involved in the regulation of cell cycle. In the CDK-independent mechanism, CCND1a and CCND1b regulate cell cycle by inducing ER/AR-mediated transcription in different manners.

Alternative splicing of CCND1 pre-mRNA is one of the oncogenic splicing events and is closely associated with the dysregulated cell cycle in cancer cells [129]. Currently, the function of CCND1 isoforms has been extensively studied, and it is generally believed that CCND1 isoforms can affect cell cycle progression through both CDK-dependent and CDK-independent mechanisms (Fig. 3). The mechanism of CCND1a and CCND1b regulating cell cycle in cancers are summarized (Table 4). In the CDK-dependent mechanism for cell cycle, CCND1a can form an active CCND1a-CDK4/6 complex by binding to and activating the G1-phase-specific CDK4/6, resulting in the phosphorylation of G1-phase cycle inhibitor protein (RB). The phosphorylated RB protein is dissociated from the bound transcription factor E2F1 to initiate transcription, which drives cells from G1 to S phase and accelerates cell proliferation (Fig. 3) [130]. Zeng et al. demonstrated that CCND1a and CCND1b can promote cell proliferation by accelerating cell cycle progression in liver cancer Huh-7 and LO2 cell lines in vitro [26]. Kim et al. investigated the effects of CCND1b small interfering RNA (siRNA) in bladder cancer SBT31A and T24 cell lines in vitro and in vivo and found that low expression of CCND1b inhibited the G1-S transition and suppressed cells proliferation [131]. This indicates that CCND1b may have the same effect as CCND1a on the CDK-dependent cell cycle mechanism, and both of them can promote cell cycle progression. However, CCND1b has revealed unexpected disparities in cell cycle regulation. Wang et al. found that overexpression of CCND1b initiated cell cycle arrest and induced apoptosis in cervical cancer HeLa cell line in vitro and in vivo, thereby inhibiting cell proliferation [129]. The reason for this difference may be related to the effect of CCND1b on RB phosphorylation. A lot of studies have shown that although CCND1b could bind to CDK4, it was significantly deficient in inducing RB phosphorylation [31, 105, 132, 133]. Thus, CCND1a and CCND1b may have different biological functions in regulating cell cycle in the CDK-dependent cell cycle mechanism. At present, the mechanism of CCND1b in cell cycle is still unclear. Therefore, how CCND1b regulates the cell cycle progression of cancer cells in a CDK-dependent manner needs to be further explored. In addition to the classical CDK-dependent cell cycle regulating activity, CCND1 itself has CDK-independent effects. CCND1 has been reported to promote cell cycle progression by regulating transcriptional factors and transcriptional coregulators involved at different levels in the cell cycle control, such as ER, androgen receptor (AR), peroxisome proliferator-activated receptor-γ (PPAR γ), SRC1, AIB1, GRIP1, STAT3 and TAFII250 [33, 134–136]. Zwijsen et al. revealed that CCND1a could substitute estrogen to activate ER-mediated transcription and contributed to estrogen-induced cell proliferation in estrogen-responsive tissues [34]. When ER is activated by estrogen, activation function domain-2 (AF-2) is exposed that specifically binds to steroid receptor coactivator-1 (SRC-1) [137–139]. The recruitment of SRC-1 to ER enhances the binding of the receptor to estrogen-responsive element (ERE), triggering transcription of target gene [107, 137]. The LxxLL motif at positions 254-259 of CCND1a has a similar structure to the AF2 of ER [35]. Therefore, CCND1a can act as a bridging factor to recruit SRC-1 into ER and activate ER-mediated transcription to promote cell cycle progression in breast cancer MCF-7 or T47D cell lines in vitro [34, 35]. However, Zhu et al. found that CCND1b could not induce ER-mediated transcription because it is unable to recruit SRC-1 to the ER [35]. Moreover, the study suggested that CCND1b could inhibit breast cancer cell proliferation by antagonizing the effect of CCND1a on ER-mediated transcription (Fig. 3). In addition, Burd et al. demonstrated that CCND1b had a different transcriptional regulation function from CCND1a in prostate cancer cell line in vitro and in vivo [140]. CCND1a was considered as a key AR corepressor [141–143]. CCND1a transcriptional regulation of the AR was manifested by discrete mechanisms. Transcriptional repression mediated by CCND1a binds histone deacetylase 3 (HDAC3) in the androgen-responsive element (ARE) region, thereby limiting androgen-dependent proliferation. Although CCND1b retains the function of AR binding, it selectively impairs the regulation of AR. In particular, CCND1b show impaired AR corepressor activity on the prostate-specific antigen (PSA) promoter. This defect causes CCND1b to stimulate androgen-dependent proliferation, in contrast to the inhibition of cell cycle progression mediated by CCND1a (Fig. 3) [140]. Thus, these studies suggest that the aberrant splicing isoforms play different roles in cell cycle regulation and may be therapeutic targets.

**Table 4:** Aberrant CCND1 splicing in cancers and its clinical application.

	Cancer type	CCND1b/a	Related function	Vitro/Vivo	Cell line	Ref
Cell cycle	Liver cancer	CCND1b↑ CCND1a↑	Promote cell cycle	Vitro	Huh-7/LO2	[26]
	Cervical cancer	CCND1b↑	Inhibit cell cycle	Vitro/Vivo	HeLa	[129]
	Bladder cancer	CCND1b↓	Suppress cell cycle	Vitro/Vivo	SBT31A/T24	[131]
	Breast cancer	CCND1b↑ CCND1a↑	Inhibit cell proliferation Expedite cell proliferation	Vitro	MCF-7/T47D	[34, 35]
	Prostate cancer	CCND1b↑ CCND1a↑	Promote cell cycle Inhibited cell cycle	Vitro/Vivo	LNCaP/PC3	[140]
Invasion and metastasis	Breast cancer	CCND1b↑	Facilitate tumor metastasis	Vitro	MCF-7/MDA-MB-231	[27]
		CCND1b↑	Promote cell invasion and metastasis	Vitro	MCF-7	[38]
		CCND1b↑	Accelerate cell invasion and metastasis	Vitro	MCF-7	[146]
	Colorectal cancer	CCND1b↑	Impel cell invasion and tumorigenesis	Vitr*o*/Vivo	293T/MEF	[144]
		CCND1b↑	Contribute to cell invasion	Vivo	/	[216]
	Bladder cancer	CCND1b↑	Stimulate cell invasion	Vitro	SBT991	[145]
		CCND1b↓	Inhibit cell invasion	Vitro	T24/SBT31A	[131]
Clinical prognosis	Breast cancer	CCND1b↑ CCND1a↑	Correlated with adverse outcomes Not associated with clinical outcome	/	/	[30]
	Thyroid cancer	CCND1b↑ CCND1a↑	Related to clinicopathologic features Associated with distant metastasis	/	/	[167]
	Non-small cell lung cancer	CCND1b↑ CCND1a↑	Related to prognosis Not related to prognosis	/	/	[168]
	Cervical cancer	CCND1b↑ CCND1a↑	Associated with lymph node metastases Related to tumor size/differentiation	/	/	[169]
	Esophageal cancer	CCND1b↑ CCND1a↑	Not correlate with overall survival	/	/	[170]
	Colorectal cancer	CCND1b↑ CCND1a↑	Not related to prognosis	/	/	[171]
Chemotherapy	Colon cancer	CCND1a↑	Resistant to doxorubicin, 5-fluorouracil	Vitro	HCT116	[39]
	Breast cancer	CCND1b↓ CCND1a↑	Sensitive to doxorubicin Resistant to paclitaxel	Vitro/Vivo	SK-BR3/MDA-MB436/Bats-72/Bads-200	[172, 173]
	Glioblastoma	CCND1a↑	Resistant to temozolomide	Vitro/Vivo	U251/SHG-44	[174]
	Liver cancer	CCND1a↓	Sensitive to 5-fluorouracil	Vitro	HepG2/SMMC-7721	[175]
	Gastric cancer	CCND1a↓	Sensitive to 5-fluorouracil	Vitro	AGS	[176]
Radiotherapy	Nasopharyngeal carcinoma	CCND1a↑	Correlated with radiosensitivity	/	/	[177]
	Esophageal cancer	CCND1a↑	Associated with radiosensitivity	Vitro	KYSE150/KYSE150R	[178]
	Oral cancer	CCND1a↑	Associated with radiosensitivity	Vitro	Ca9-22/SCC25	[179]
	Ependymoma	CCND1a↑	Associated with radioresistant	Vitro	293T	[181]
	Lung cancer	CCND1a↑	Enhance irradiation resistance	Vitro	A549/H460/293T	[182]
	Breast cancer	CCND1a↓	Increased proton radiosensitivity	Vitro	MDA-MB-231/Hs578T	[180]

### Invasion and metastasis

**Fig. 4:**
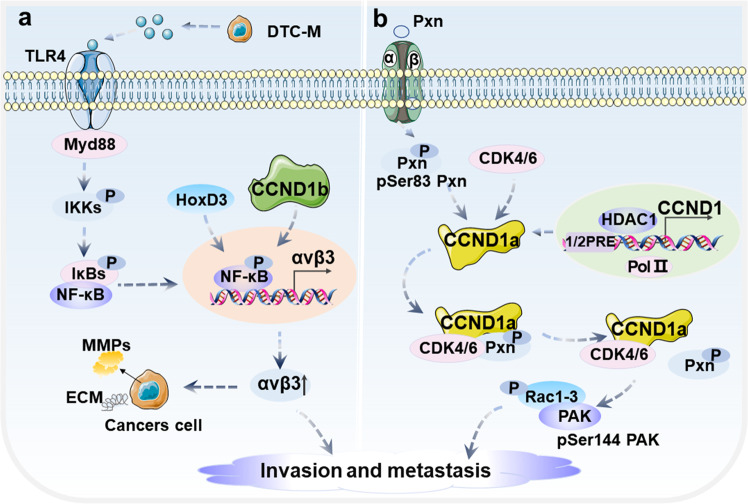
Cell invasion and metastasis regulated by CCND1 isoforms. **a** CCND1b can synergize with HOXD3 to regulate αvβ3 expression, thus enhancing cell invasion and metastasis. **b** CCND1a-CDK4/6 complex promotes the activation of small GTPase RAC1 by regulating the phosphorylation of Pxn to induce cell invasion and metastasis.

The isoforms of CCND1 had been deemed to contribute to the invasion and metastasis of various human cancers [38, 144, 145]. The mechanism of CCND1b isoform regulating cell invasion and metastasis in cancers is summarized in Table 4. CCND1b promotes invasion and metastasis in a manner independent of CCND1a in breast cancer MCF-7 cell line in vivo [38, 146]. Specifically, CCND1b can modulate the metastatic phenotype characterized by αvβ3 expression and synergize with HOXD3 to enhance the invasive and metastasis potential of breast cancer cells (Fig. 4a). αvβ3 is a key integrin mediating the invasive and migration of cancer cells [147, 148]. Interestingly, αvβ3 is overexpressed only in metastatic tumor cells, while it is very low expressed in non-metastatic cancer cells [148, 149]. It has been found that even if NF-κB is effectively activated by TLR4 ligand, the expression of αvβ3 cannot be effectively increased by NF-κB in non-metastatic MCF-7 cells [149]. In addition, CCND1b has also been found to promote cell invasion via Erk or RB phosphorylation in bladder cancer and rectal tumorigenesis [144, 145]. By introducing the CCND1b siRNA, Kim et al. demonstrated that the CCND1b siRNA significantly inhibited the invasiveness of bladder cancer cells in vivo [131]. Since CCND1b promotes invasion and metastasis of cancer cells, CCND1b can be used as a new clinical target for anti-metastasis therapy. However, different from the mechanism of CCND1b, CCND1a may participate in the process of cell invasion and metastasis through various mechanisms [1]. Studies have reported the mechanism of a new regulatory axis composed of CCND1-CDK4/Paxilin-Rac1 in the invasion and metastasis of cancer cells [1, 150, 151]. Paxillin (Pxn) is a key component of local adhesion for the monitoring of Rho GTPases [152]. Pxn has many phosphorylation sites, and the CCND1a-CDK4 complex may promote the activation of small GTPase RAC1 by regulating the phosphorylation of Pxn to induce cell invasion and metastasis (Fig. 4b) [1, 151]. Although a lot of studies have shown that CCND1 subtype is related to the invasion and metastasis of cancer cells, how CCND1 isoforms regulate invasion and metastasis independently from the cell cycle still needs to be further explored.

## CCND1B transgenic mice models

The CCND1 transgenic mice model has been widely used to find new biological functions of CCND1 and contribute to better understand the role of CCND1 in tumorigenesis in vivo [153, 154]. A large body of literature has shown that genetic alterations of *CCND1* are extremely common in human cancers [155]. However, in vitro functional studies have not exhibited transforming activity and CCND1 overexpression has shown weak or no carcinogenic activity in vivo transgenic models [156–159]. Rodriguez-Puebla et al. confirmed that CCND1 overexpression in mice epidermis can increase cell proliferation and the activity of the cyclin-dependent kinase in vivo, but it does not affect the development of skin tumor by transgenic mice (K5D1 mice) overexpressing CCND1 [157]. Robles et al. also agreed with this view, believing that the expression of CCND1 in epithelial tissues of transgenic mice resulted in epidermal hyperproliferation and severe thymic hyperplasia, which were not related to the development of skin tumors [158]. Yet, studies with CCND1-null mice (D1KO) or CCND1 deficient cells have indicated that CCND1 is necessary for tumor development [160–163]. This paradox may be related to the existence of alternative splice product of CCND1. By exploring transgenic mice expressing human CCND1b under the control of the bovine K5 promoter (K5D1b mice), Rojas et al. found that K5D1b mice basically had no macroscopic or microscopic phenotype. Interestingly, the skin carcinogenesis of K5D1b mice was enhanced and lack of thymus hyperplasia [164]. The lack of thymus phenotype in K5D1b mice may be related to the functional loss of exon 5 in CCND1b [165]. To elucidate the carcinogenic potential of CCND1b, Kim et al. developed CCND1b transgenic mice and indicated that CCND1b expression was conducive to female-specific rectal carcinogenesis. In addition, Augello et al. described the first-in-field model for switching from CCND1a to CCND1b using a new genetically engineered mice model. It provided the first genetic evidence for CCND1b as an oncogene [166]. Notably, this study not only confirmed the first preclinical evidence for the method of specifically targeting CCND1b-expressing tumors, but also provided the basic principle for developing CCND1b expression as a new biomarker of therapeutic response. Thus, the transgenic mice model of CCND1b is a favorable model for studying the role of CCND1b in cancer, which is essential for preclinical research.

## Clinical impaction of CCND1 isoforms in cancer

### Clinical prognosis

CCND1 isoforms are associated with disease risk and/or clinical outcome in cancers, and can be used to some extent to predict cancer risk, clinical prognosis, or therapeutic response (Table 4). Multiple clinical studies have suggested that CCND1b overexpression could be used as a prognostic marker in breast cancer, non-small cell lung cancer, and thyroid cancer [30, 167, 168]. Millar et al. showed that high expression of CCND1b was independently relevant to adverse outcomes in breast cancer, including recurrence, metastasis, and decreased survival [30]. However, the high expression of CCND1a was inversely associated with Ki67 markers and not correlated with clinical prognosis [30]. Since CCND1b functions independently of CCND1a, the association between CCND1b and disease outcome is not regulated by the state of CCND1a. Abramson et al. analysed CCND1a and CCND1b expression in primary human breast cancer and found that patients who co-expressed CCND1a and CCND1b had a higher risk of recurrence than the expression of either alone [37]. Additionally, the expression of nuclear CCND1b in papillary thyroid cancer was associated with aggressive clinicopathological features, including lymph node metastasis, risk of recurrence, and advanced stage, while the expression of cytoplasmic CCND1b was related to lymph node metastasis and high risk for cancer recurrence [167]. In non-small cell lung cancer, CCND1b expression was associated with prognosis, whereas CCND1a expression was not relevant to prognosis [168]. In cervical cancer, CCND1a expression was connected to tumor size and degree of differentiation, and CCND1b expression was associated with lymph node metastasis, but it was not related to the prognosis of cervical cancer [169]. Moreover, Gupta et al. indicated that although increased total CCND1 expression was correlated with survival in esophageal adenocarcinoma, the expression of CCND1a and CCND1b was not associated with overall survival [170]. In colorectal cancer, the expression of CCND1a and CCND1b was also not significantly related to prognosis [171]. These studies indicate that CCND1b plays a vital role as a clinical prognosis marker in cancers and warrants further investigation.

### Chemotherapy

**Fig. 5:**
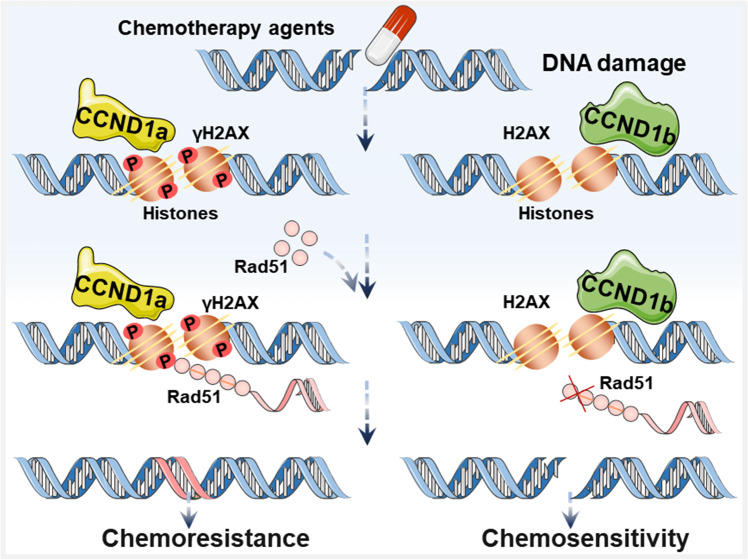
CCND1 isoforms can regulate resistance to DNA damage therapy by inducing DNA damage response in cancer. CCND1a increased the phosphorylation of γH2AX induced by chemotherapy agents and recruited Rad51 to local chromatin in response to DNA damage, leading to chemoresistance. CCND1b failed to recruit Rad51 even in response to DNA damage, resulting in chemosensitivity.

Chemoresistance is currently obstructing the success of chemotherapy in cancer. CCND1 isoforms have been shown to contribute to clinical responses and provide therapeutic targets for chemotherapeutic drugs [39, 172]. Plenty of evidence suggested that the expression of CCND1a and CCND1b was related to chemoresistance in cancers (Fig. 5) [39, 173]. In Table 4, we summarize the role of two CCND1 isoforms in modulating chemoresistance in cancers. A study on colon cancer revealed that CCND1a increased the phosphorylation of γH2AX induced by chemotherapy agents (doxorubicin or 5-fluorouracil) and recruited Rad51 to local chromatin in response to DNA damage, triggering DDR characterized by phosphorylation of γH2AX in colon cancer HCT116 cell line in vitro. However, CCND1b failed to recruit Rad51 even in response to DNA damage [39]. Regarding breast cancer, the CCND1b siRNA has been demonstrated to synergistically enhance the cell killing effect of doxorubicin, thereby inhibiting tumor growth in mice model [173]. CCND1a was also found to mediate paclitaxel resistance of breast cancer cells through the pRB/E2F1 pathway and AKT phosphorylation [172]. Additionally, Myklebust et al. identified the upregulation of CCND1a expression as a positive predictor of adjuvant 5-fluorouracil and levamisole therapy for colon cancer, especially in stage III colon cancer [171]. Upregulated CCND1a expression was also proved to be involved in chemoresistance to temozolomide in human malignant glioma cells [174]. Consistently, induction of CCND1a silencing in liver cancer HepG2 and SMMC-7721 cell lines significantly increases susceptibility to 5-fluorouracil in vitro [175]. Similarly, this phenomenon had also been observed in gastric cancer AGS cell line [176]. Therefore, the expression of CCND1a and CCND1b during cancer treatment is crucial for the proper selection of chemotherapeutic drugs.

### Radiotherapy

The splicing favor of CCND1a is associated with radiosensitivity (Table 4). Fu et al. analysed the expression of CCND1a in biopsy specimens of nasopharyngeal carcinoma patients by immunohistochemical method and found that the expression level of CCND1a was negatively associated with the radiosensitivity of nasopharyngeal carcinoma [177]. Additionally, the high expression of CCND1a improved the sensitivity of radiotherapy for oral squamous cell carcinoma and esophageal cancer in vitro [178, 179]. Moreover, Choi et al. demonstrated that low expression of CCND1a increased proton radiosensitivity in triple-negative breast cancer cells in vitro, possibly because depletion of CCND1a prevents Rad51 recruitment to double-strand break sites [180]. It has been reported that DNA repair induced by upregulated CCND1a expression demonstrated a potential radioresistant mechanism in ependymoma 293T cell line in vitro [181]. A recent study also showed that upregulated CCND1a expression could promote DNA repair and enhance radioresistance in lung cancer cells in vitro [182]. However, no association of CCND1b expression with radiosensitivity was observed. Therefore, these findings suggested that CCND1a is closely related to the radiosensitivity of cancer cells and may play a vital role in the regulation of cellular radiosensitivity.

## Strategies modulating CCND1 isoforms in cancer

### Antisense oligonucleotides

**Fig. 6:**
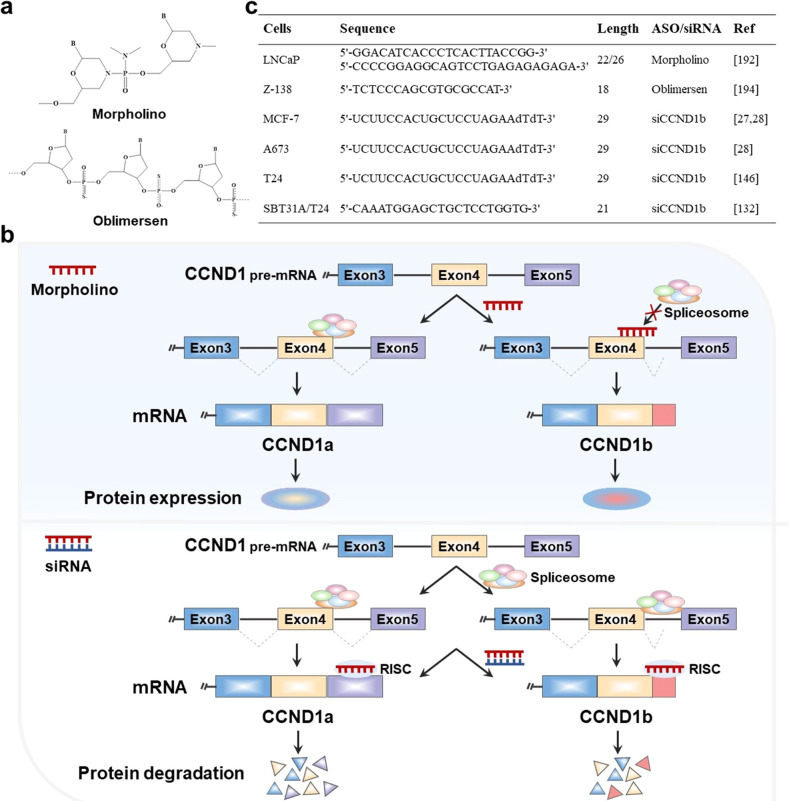
Strategies modulating CCND1 isoforms in cancer. **a** Chemical formulae of morpholino and oblimersen. **b** Morpholino that binds to the exon 4/intron 4 boundary prevents the binding of spliceosome, resulting in a splicing shift to the short isoform CCND1b. siRNA targeting CCND1 isoforms can down-regulate the expression of CCND1 isoforms by binding to the mRNA of target gene. **c** Sequences of ASO and siRNA used to modulate CCND1 isoforms.

Small molecule modulators of pre-mRNA splicing represent an attractive option for establishing novel therapeutic strategy in cancer treatment [183]. To date, antisense oligonucleotides (ASO) have been widely used to modulate the splicing mode of pre-mRNA. ASO, typically 15–30 nucleotides, is a short, synthetic, antisense, and modified single-stranded deoxyribonucleotide analogue [184]. It is designed to base pairs in an antisense orientation to a specific pre-mRNA sequence and create a steric hindrance to the binding of splicing factors to the pre-mRNA [185]. This binding alters the recognition of splice sites by the spliceosome, ultimately leading to splicing isoforms switching. Morpholino antisense oligonucleotides (MAOs) are the third-generation ASO, whose ribose is replaced by a morpholino ring and the negatively charged phosphodiester bond is substituted with a neutral phosphoramidate linkage (Fig. 6a) [186]. It has been extensively used to modify splicing and can completely and specifically block splicing events [187–190]. In general, MAO can base pairs with alternative splice site of CCND1 pre-mRNA to block the aggregation of spliceosome and the binding of splicing factors to their target sequences, which lead to the splicing favorable switch [191]. Since the CCND1b transcript is generated due to a failure to splice at the exon 4/intron 4 boundary of CCND1 pre-mRNA, Augello et al. inhibited splicing at the exon 4/intron 4 boundary by designing an MAO that specifically binds to CCND1 mRNA [191]. Although MAO treatment did not affect overall CCND1 levels, the introduction of MAO into prostate cancer cells effectively suppressed the splicing events of CCND1a and upregulated CCND1b transcripts in a dose-dependent manner, and eventually led to increased prostate cancer cell proliferation and invasion (Fig. 6b). In addition, the study has confirmed that the level of CCND1a is down-regulated by miR-195 overexpression. Zhang et al. designed an ASO against miR-195, which could reduce the level of miR-195 by binding to endogenous miR-195, thereby upregulating CCND1a expression and eventually leading to the proliferation of colorectal cancer cells in vitro [192]. Moreover, a related study found that Bcl-2 silencing was associated with decreased CCND1a expression but not with CCND1b in mantle cell lymphoma cell lines in vitro [193]. Therefore, the use of ASO against Bcl-2 (oblimersen) can also indirectly inhibit the transcription of CCND1a through the interaction between Bcl-2 and CCND1a, resulting in higher proliferation and invasive of mantle cell lymphoma. Figure 6c summarizes some sequences of ASO related to the regulation of CCND1 pre-mRNA splicing. In a word, ASO is an effective strategy to correct the expression of cancer-related CCND1 isoforms through redirection of splicing and rebalancing the ratio of CCND1a/CCND1b.

### Small interfering RNA

In addition to ASO, RNA interference (RNAi) is the most common transcript-targeted therapy tool that can be used to modulate gene expression [194]. RNAi, the process of machining double-stranded RNA into short siRNA, has been used to target mRNA to downregulate gene expression via degradation of Dicer-RNA induced silencing complex pathway (RISC) [195]. Compared to ASO technologies, RNAi relies on a catalytic mechanism, because after cleavage of the target mRNA, siRNA-loaded RISC can isolate and link to another mRNA molecule, which mainly depends on 100% complementarity of the bind [196, 197]. Therefore, extremely low siRNA concentrations are able to induce efficient target gene knockdown [198]. siRNAs are also widely used to modulate the expression of aberrant isoforms. Some siRNA sequences targeting CCND1b used in studies have been summarized in Fig. 6c. Sanchez et al. efficiently decreased the expression of CCND1b by using the siRNAs that specifically bind to CCND1b, resulting in reduced tumorigenesis in Ewing sarcoma cells in vitro [28]. In general, after the siRNA targeting CCND1a and CCND1b enters the cell, it is integrated to form RISC under the guidance of its antisense strand. The endogenous mRNA with homologous sequences is cleaved by binding to RISC, ultimately leading to the silencing of CCND1a and CCND1b (Fig. 6b). In addition, Kim et al. demonstrated the use of siRNA targeting CCND1b would be a novel therapy for CCND1b-expressing in bladder cancer cell line in vitro. It suppresses the malignant phenotypes of bladder cancer by inducing apoptosis, inhibiting the cancer cell stemness, and epithelial-mesenchymal transition [131]. Moreover, CCND1b can promote the invasion and metastasis of breast cancer cells in vitro, and the use of siRNA targeting CCND1b may provide a new target for the treatment of metastatic breast cancer [27]. Currently, siRNAs have been identified as therapeutic tools for the treatment of cancers [199]. Therefore, siRNA targeting CCND1b is also expected to develop into new anti-cancer drugs.

## Conclusions

*CCND1* has many mutation types with different mutation frequencies in human cancers. The G870A mutation has an extremely high probability of mutation in clinical tumor and is closely related to the risk, treatment, and prognosis of multiple cancer. The G870A mutation is widely regarded as an attractive target for clinical prediction in cancers. However, how the G870A mutation is generated and its molecular mechanism in cancer need to be further explored. Gene expression disorder caused by abnormal RNA splicing is also an important reason for the genesis and development of cancer [200, 201]. Currently, the regulatory of splicing has become a potent therapeutic strategy for cancer. Studies have proved that the production of CCND1a and CCND1b by alternative splicing of G870A mutation plays a key role in the occurrence and development of cancer. It is generally believed that the imbalanced CCND1a/b ratio can cause cancer, and the high expression of CCND1b is closely related to carcinogenicity. In recent years, in vitro and in vivo studies have revealed the new effect of CCND1b in cell cycle, cell invasion, and metastasis. Moreover, the internal relationship between CCND1b-regulated cell invasion and metastasis has been discovered, providing a new clinical viewpoint for targeting CCND1b anti-metastasis therapy. However, the biological mechanism of preferential splicing of CCND1b results in its high expression in cells is not explicit. Therefore, it is still urgent to further study the mechanisms of CCND1 isoforms in cells. Correction of CCND1 splicing by ASO and small molecule modulators has been shown efficacy in cancer therapy. The development of splicing regulatory drugs targeting CCND1b is expected to become a new option for cancer treatment. Nevertheless, an inhibitor of specific splicing factor for CCND1 splice correction needs to be identified. Besides CCND1, the cyclin family members including CCNA [202], CCNB [203], and CCNE [204] also have multiple splice isoforms, but it is not clear that the multiple splice isoforms of the other family members play a finely coordinated biological role. Thus, there still exists much work in future research on this gene.

## Data Availability

All data generated or analyzed during this study are included in this published article.
